# Nitrogen Plasma Treatment of Composite Materials Based on Polylactic Acid and Hydroxyapatite

**DOI:** 10.3390/polym16050627

**Published:** 2024-02-25

**Authors:** Ulyana V. Khomutova, Alena G. Korzhova, Anastasia A. Bryuzgina, Olesya A. Laput, Irina V. Vasenina, Yuriy H. Akhmadeev, Vladimir V. Shugurov, Ivan I. Azhazha, Yelena G. Shapovalova, Aleksandr V. Chernyavskii, Irina A. Kurzina

**Affiliations:** 1 Chemical Department, National Research Tomsk State University, 36 Lenin Ave., Tomsk 634050, Russia; ugoroshkinau@gmail.com (U.V.K.); korzhova17@gmail.com (A.G.K.); bryuzgina2016@mail.ru (A.A.B.); egshapovalova@mail.tsu.ru (Y.G.S.); kurzina@mail.tsu.ru (I.A.K.); 2 P.N. Lebedev Physical Institute, 53 Leninsky Prospekt, Moscow 119333, Russia; ivpuhova@mail.ru; 3 Institute of High Current Electronics, 2/3 Akademichesky Ave., Tomsk 634055, Russia; ahmadeev@opee.hcei.tsc.ru (Y.H.A.); shugurov@opee.hcei.tsc.ru (V.V.S.); vanya.azhazha@gmail.com (I.I.A.); 4 Nanocenter MIREA, MIREA—Russian Technological University, 78 Vernadskogo Ave., Moscow 119454, Russia; chernav@yahoo.com

**Keywords:** polylactic acid, hydroxyapatite, composite materials, surface modification, plasma treatment, arc discharge, chemical compound, wettability, biocompatibility, cell viability

## Abstract

The effect of surface modification by an arc discharge plasma in a nitrogen flow with treatment durations of 5 and 10 min on the physicochemical properties and biocompatibility of the surface of composites based on polylactic acid and hydroxyapatite (PLA/HA) with different mass ratios (80/20, 70/30, 60/40) has been investigated. The aim of this work was to show the correlation between the changes of the physicochemical characteristics (chemical compound, morphology, wettability) of the surface layer of the PLA/HA composites and the cell viability (macrophages) in the presence of the plasma-modified materials. The dependence of alterations of the functional properties (wettability, biocompatibility) on the change in the chemical composition under the plasma exposure has been established. The chemical composition was studied using X-ray photoelectron spectroscopy (XPS), the surface morphology was researched with scanning electron microscopy (SEM), and the wettability of the composite’s surface was analyzed by measuring the contact angle and surface energy calculation. In addition, the viability of macrophages was investigated when the macrophages from three donors interacted with a modified PLA/HA surface. It was found that the formation of the new functional groups, –C-N and N-C=O/C=O, improves the wettability of the surface of the composites and promotes the viability of macrophages in the presence of the composite materials. The fundamental principles for obtaining promising materials with the required properties for eliminating bone defects have been created.

## 1. Introduction

Currently, restoration of bone tissue defects due to injuries, fractures, and age-related changes is an urgent problem in regenerative medicine, since there is always a high risk of developing inflammatory processes in bone tissue, as well as in adjacent soft tissues [1]. In this regard, interest in obtaining artificial materials capable of restoring and replacing bone tissue has sharply increased. The high cost, low biocompatibility, and bioactivity as well as unsatisfactory mechanical characteristics [2] of the bone substitutes used do not allow a quick solution to this problem. The optimal solution to this problem is the creation of materials that could not only temporarily replace sections of bone tissue, but also promote its restoration without the risk of repeated operations [3].

Materials based on calcium phosphates and biodegradable polymers are of the greatest interest. Composite mixtures of these materials may obtain unique functional properties, such as high restorability, sufficient mechanical strength, and the absence of negative reactions from the living body [4,5,6]. Composite materials based on polylactic acid and hydroxyapatite (PLA/HA) were recognized as the most promising biocompatible materials for bone implantation, since polymer based on lactic acid can decompose in a living body without the formation of toxic compounds, while the inorganic component—hydroxyapatite (HA)—is the main mineral component included in natural human bone tissue [7]. Moreover, HA has osteoconductive properties and a low resorption rate, so it can be widely used as a bone graft material [8,9]. 

The production of composites by combining PLA and HA has been the subject of recent research. The purpose of these studies is to improve the adhesion of the constituent phases in the boundary layers and increase the mechanical strength of the material [10]. It was previously found [11] that the mass ratios of polylactic acid/hydroxyapatite (PLA/HA) components of 80/20, 70/30, and 60/40 are optimal for obtaining materials with high functional properties. The main disadvantage of these materials is the absence on the surface that comes into contact and interacts with the biological environment of carboxyl and nitrogen-containing and other functional groups [12]. Due to the hydrophobic surface and, accordingly, the low value of free surface energy, effective attachment and proliferation of cells on the surface of polylactic acid and PLA/HA composite materials does not occur. This problem can be solved by exposing the surface of the composite to low-temperature plasma in a nitrogen flow [13,14].

In recent years, modification of the surface of polymers and composites based on radiation techniques has become a popular trend. Under conditions of energy exposure, complex physicochemical processes are initiated in the surface layers of materials that can significantly change the structural and functional properties of their surface [15]. It is known that plasma modification affects the physicochemical and functional characteristics of the surface without changing the bulk properties of the materials [16].

The most important property in determining the practical application of composite materials in medicine is wettability, which is related to roughness, adhesion, and surface chemistry [17]. Plasma treatment is an effective method for surface modification of polymer and composite materials in terms of surface activation and improved biocompatibility [18,19,20]. Low-temperature plasma treatment of polymers leads to breaking of the polymer chain, accompanied by oxidation of the surface and the formation of new functional groups, which increase the hydrophilicity of the material [21]. The use of nitrogen as a plasma-forming gas promotes the appearance of various hydrophilic functional groups, including amine (NH_2_, NH), imine (C=N), and amide (O=C–N), which, along with cleaning the surface from contaminants under the influence of plasma, lead to improved adhesion properties of the surface of polymers and composites based on them and provide a suitable substrate for cell attachment [22]. Polar groups are known to bind biologically active molecules to which cellular receptors attach, thereby initiating proper interactions between the cell and the material [23]. 

Numerous studies have shown that treatment of PLA-based materials with low-temperature nitrogen plasma effectively changes the surface wettability from a hydrophobic to a hydrophilic state and significantly improves the affinity of the surface for various types of cells [24]. Therefore, the study of physicochemical processes occurring in materials treated with plasma flows, as well as studies of the influence of the plasma treatment conditions on changes in functional properties, chemical composition of the surface, stoichiometry of HA in the composite, and biological properties are promising directions. The purpose of this work was to study the effect of surface modification by arc discharge plasma in a nitrogen flow with treatment durations of 5 and 10 min on the physicochemical properties and biocompatibility of the surface of composites based on polylactic acid and hydroxyapatite (PLA/HA) with different mass ratios (PLA/HA = 80/20, 70/30, 60/40).

## 2. Materials and Methods

### 2.1. Preparation of the Experimental Samples

**Figure 1:**
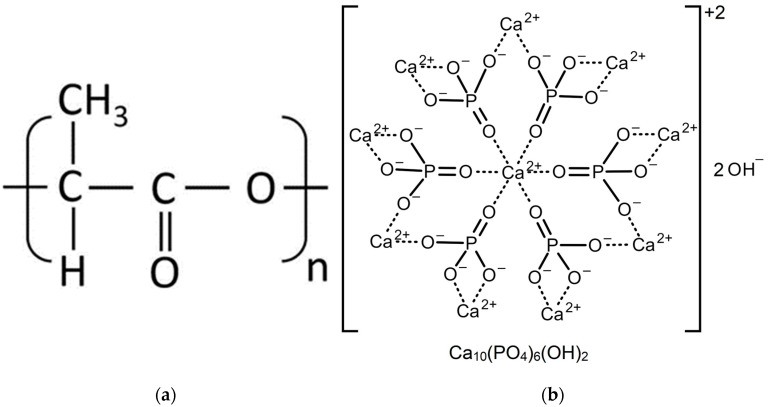
Structural formulas: (**a**) PLA; (**b**) HA.

**Figure 2:**
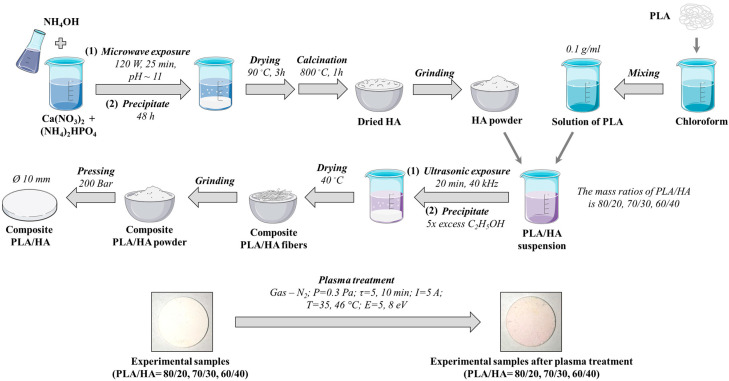
Scheme for obtaining the PLA/HA composites. The figure was partly generated using Servier Medical Art, provided by Servier, licensed under a Creative Commons Attribution 3.0 unported license.

In this work, composite materials based on polylactic acid and hydroxyapatite (PLA/HA) were studied. The structural formulas of PLA and HA are shown in Figure 1. The preparation of the PLA/HA samples was carried out according to a previously proposed scheme [11] in the laboratory of the National Research Tomsk State University (NR TSU, Tomsk, Russia). Polylactic acid was obtained from *l*-lactid initial monomer synthesized in advance in the Laboratory of Polymers and Composite Materials at Tomsk State University according to the technique described in [25,26]. When synthesized and dried, the PLA is a ball of fibers, as one can see in the right upper corner of Figure 2. Hydroxyapatite synthesis was carried out using the liquid-phase method with microwave radiation at pH ~11 according to the patented technology [16,27] by the following equation:

10Ca(NO_3_)_2_ + 6(NH_4_)_2_HPO_4_ + 8NH_3_∙H_2_O → Ca_10_(PO_4_)_6_(OH)_2_ + 20NH_4_NO_3_ + 6H_2_O

For obtaining the experimental composites PLA/HA, a polylactic acid solution was mixed with a hydroxyapatite powder under constant stirring. The mass ratios of the components were 80/20, 70/30, and 60/40 of the PLA and HA, respectively. The obtained suspension was ultrasonically treated for 20 min at a frequency of 40 kHz, then precipitated in a fivefold excess of ethyl alcohol. The precipitation process involves pouring the composite solution in a thin stream into a fivefold excess of ethyl alcohol, resulting in the formation of a ball of fibers. The obtained fibers were then dried until complete evaporation of the solvent in a desiccator at 40 °C. The obtained composite materials were mechanically pulverized, then molded on a laboratory hydraulic press at a pressure of 200 bar. The diameter of the obtained tablets was 10 mm. The general scheme of the sample preparation is presented in Figure 2. It should be mentioned that in the obtained composite materials, the polymer component (polylactic acid), and the inorganic component (hydroxyapatite) do not form chemical bonds with each other, i.e., only weak interaction forces are between them.

### 2.2. Study Design

**Figure 3:**
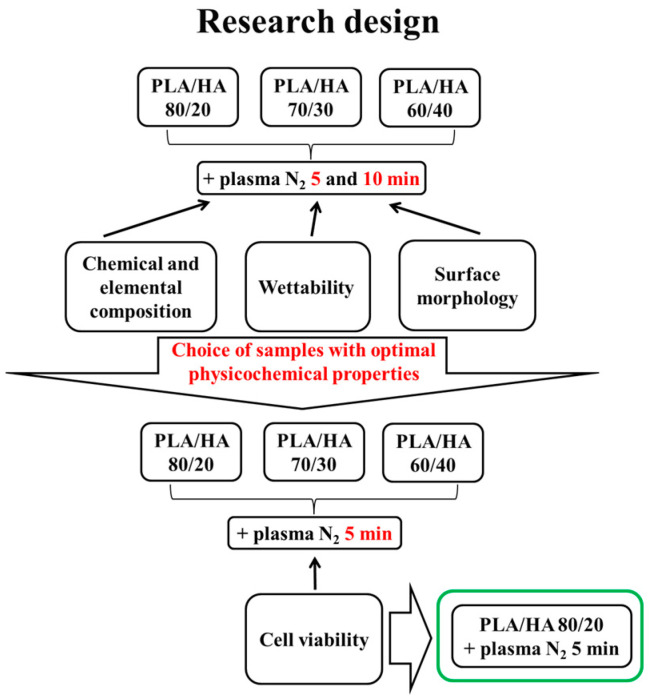
Study design.

According to our previous results of the PLA scaffolds’ arc discharge plasma treatment in nitrogen flow [28], the PLA/HA 80/20, 70/30, and 60/40 composites were treated at similar plasma exposure parameters. The chemical and elemental composition, wettability, and surface morphology of the experimental samples were investigated. Based on the results, the PLA/HA composite samples with optimal physicochemical properties for further cell research were selected. The mode of the 5 min plasma treatment revealed the most significant effect on the change in the physicochemical characteristics of the PLA/HA surface. The viability of macrophages was evaluated in the presence of the 5 min nitrogen plasma-treated PLA/HA composites. The study design is summarized in Figure 3.

### 2.3. Plasma Treatment of PLA/HA Composites

The processing of the experimental samples PLA/HA 80/20, 70/30, and 60/40 was carried out using a PINK plasma generator [29] based on an arc discharge in a nitrogen flow in the Laboratory of Plasma Emission Electronics of the Institute of High Current Electronics of the Siberian branch of the Russian Academy of Sciences (HCEI SB RAS, Tomsk, Russia). The two modes of plasma treatment, differing in the duration of exposure (5 and 10 min), were used. The resulting plasma was evenly distributed throughout the working chamber where the PLA/HA samples were located. Table 1 shows the parameters of the plasma treatment.

### 2.4. Characterization Techniques

#### 2.4.1. Chemical Composition

The elemental composition of the surface was studied by X-ray photoelectron spectroscopy (XPS) using a PhiX tool-automated XPS microprobe with a KαAl source at the Nanocenter of the Moscow State University of Information Technologies, Radio Engineering and Electronics. The samples were mounted on a holder using conductive carbon or copper tape. For the XPS analysis, a monochrome X-ray source with an X-ray spot size of 400 μm^2^ was used. During the analysis, a standard charge compensation system with low electron and ion energy (~0.1 eV) was used. Further analysis of the elemental composition was carried out using the Casa XPS program [16]. The calculation of the atomic content of the elements was carried out using the characteristic factors of relative sensitivity. To study the chemical environment of the atoms on the surfaces of the PLA/HA samples, the high-resolution spectra were deconvoluted. The approximating line shapes, peak half-widths, and binding energies corresponding to the peak maximum were determined from the spectra of the PLA/HA samples. The Tougaard background was used for noise subtraction. To construct a mathematical model of the spectra, the Gaussian–Lorentzian function was applied to the elementary components.

#### 2.4.2. Surface Morphology

The surface morphology was studied using a Quanta 200 3D scanning electron microscope (SEM) and focused ion beam instrument at the Tomsk Materials Science Center for Collective Use at an accelerating voltage of 5–20 kV with a spot size of 40 μA/μm^2^. Before the SEM study, the samples were coated with a conducting graphite film of 2–5 nm thickness using magnetron sputtering to alleviate charge buildup on the surfaces.

#### 2.4.3. Wettability

The water and glycerol contact angles were measured using a sessile drop technique using a Kruss Easy Drop (DSA25) instrument (KRÜSS, Hamburg, Germany) with DSA1 software (NR TPU, Tomsk, Russia). The surface energy calculation was carried out using the Owens–Wendt–Rabel–Kjelble equation [30].

#### 2.4.4. Cell Viability Assay

A biocompatibility study was performed using primary monocytic-derived macrophages as a model system. Monocytes were isolated from the peripheral blood of three healthy donors according to a previous method [31]. Cultivation of the monocytes at a concentration of 10^6^ cells/mL was carried out in X-VIVO 10 medium (Lonza, Antwerp, Belgium) supplemented with 1 ng/mL M-CSF (Peprotech, Germany) and 10^−8^ M dexamethasone (Sigma-Aldrich, Darmstadt, Germany). The samples were completely immersed in the culture medium with cells in the wells of the culture plate and incubated at 37 °C and 7.5% CO_2_ for 6 days. The monocytes cultured without adding samples were used as a positive control.

All the samples were previously sterilized for 30 min in 70% ethanol and for 20 min under UV light. After 6 days of culturing the monocytes in the presence of the composites, a cytotoxicity analysis was carried out using the alamarBlue^TM^ reagent (Thermo Fisher Scientific, Waltham, MA, USA). The reagent was added to each well at a rate of 10 µL per 100 µL of medium with cells, then the cells with alamarBlue^TM^ were incubated for 3 h at 37 °C and 7.5% CO_2_. The cells cultured without composites were used as a positive control; X-VIVO 10 culture medium with the addition of alamarBlue^TM^ reagent in a ratio of 10/1 was used as a negative control. After incubation, 100 μL of liquid was taken from each well and placed in triplicate into a 96-well plate. An Infinite 200Pro automated microplate analyzer (Tecan, Männedorf, Switzerland) was used to determine the intensity of the absorption signal at a wavelength of 570 nm.

Cellular studies were carried out in the Laboratory of Translational Cellular and Molecular Biomedicine of the Tomsk State University (Tomsk, Russia) and the Oncology Research Institute of the Tomsk Research Medical Center (Tomsk, Russia).

#### 2.4.5. Statistics

The data were subjected to statistical analysis using a two-tailed Student’s *t*-test. *p*-values of <0.05 were considered statistically significant. All the data presented are expressed as mean ± standard deviation. For each research method, at least three measurements of each sample were carried out, namely, the XP spectra were taken at three points for each sample; five spots of each sample with different magnification (from 1000× to 20,000×) were scanned during the SEM study; three sessile drops were placed for contact angle measurements; and the cells of three donors were used to cover each of the selected samples for the viability assay.

## 3. Results and Discussion

### 3.1. Elemental and Chemical Composition of the PLA/HA Surface before and after Nitrogen Plasma Treatment

Changes in the elemental and chemical composition and surface morphology under plasma treatment conditions affect the functional properties of composite materials based on polylactic acid and hydroxyapatite (PLA/HA), which determine their effectiveness for further use in biomedicine, namely, in bone implantology.

Previously, the effect of changes in the physicochemical characteristics of the PLA scaffolds under conditions of the 5-, 10-, 20-, and 30-min low-temperature nitrogen plasma treatment on the biological properties of the material was investigated [28]. The appearance of new peaks in the XPS spectra—atomic nitrogen (with a binding energy of ~399.9 eV) and the –C-N bond (with a binding energy of 286.4 eV)—was established. It has been shown that, because of plasma exposure, the wettability characteristics of PLA are significantly improved, and the surface becomes hydrophilic. It can be noted that in the presence of the 5 min nitrogen plasma-modified PLA scaffold, the viability of macrophages remains at the control level. The modes with the plasma treatment durations of 5 and 10 min were discovered to be the most effective ones. Accordingly, in this work, it was decided to use these modes to carry out the surface modification of the PLA/HA composite materials.

**Figure 4:**
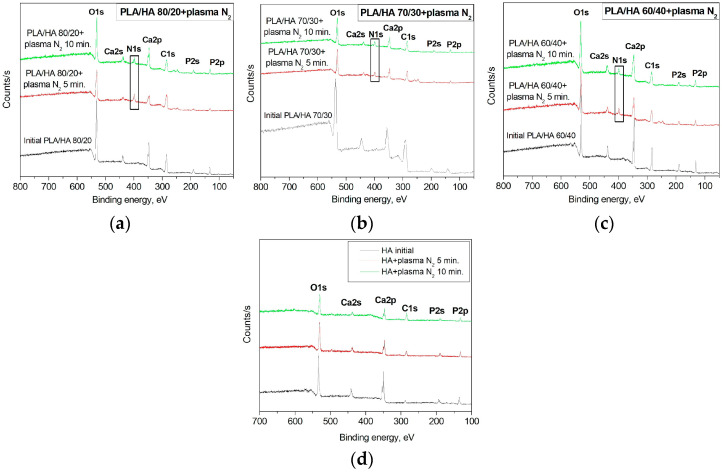
Survey XPS spectra of the (**a**) PLA/HA 80/20, (**b**) PLA/HA 70/30, (**c**) PLA/HA 60/40, and (**d**) HA before and after nitrogen treatment arc discharge plasma.

Figure 4 shows the survey XPS spectra for PLA/HA 80/20 (Figure 4a), 70/30 (Figure 4b), and 60/40 (Figure 4c) after the nitrogen arc discharge plasma treatment. These spectra reveal the presence of the main PLA/HA elements (calcium, oxygen, carbon, phosphorus), but a new peak with a binding energy of ∼399.9 eV, corresponding to atomic nitrogen, appeared following the nitrogen plasma treatment. The maximum atomic nitrogen content (12.74 ± 0.3 at. %) was observed in the surface layer of the 5 min plasma-treated PLA/HA 80/20 (Table 2). However, the higher the content of the inorganic component (HA 30% and 40%) in the composites, the less the atomic concentration of nitrogen after the plasma treatment. This is probably due to hydroxyapatite exhibiting inertness (or resistance) to surface plasma treatment in this energy range (5–8 eV), i.e., the energy of the incident particles in the plasma is not enough to penetrate the surface layer of hydroxyapatite. This is confirmed by the XPS spectra of pure HA (Figure 4d). No nitrogen peak was detected on the HA spectra after plasma irradiation. There are also no significant changes in the wettability characteristics and surface morphology. Therefore, we do not consider this material further in this paper.

We found that the carbon atomic concentration decreased after plasma modification because of the increase in the atomic oxygen concentration (Table 2). The atomic [C, at. %]/[O, at. %] ratio decreased with the nitrogen plasma treatment from 1.10 to 0.98 and 0.85 for PLA/HA 80/20; from 1.10 to 0.94 and 0.95 for PLA/HA 70/30; and from 0.98 to 0.97 and 0.96 for PLA/HA 60/40 after plasma treatment durations of 5 and 10 min, respectively. We suggest that this indicates a surface oxidation process during the plasma treatment.

The calcium–phosphate ratio [Ca, at. %]/[P, at. %] was observed to be slightly decreased in the plasma treatment conditions (Table 2). This may likely be because calcium is more susceptible to being knocked out of the structural chain when exposed to charged particles in the plasma [32]. Since changes in the stoichiometry of hydroxyapatite after plasma irradiation are insignificant, we conclude that the modification of the physicochemical characteristics of composites occurs mainly due to changes in the polymer component.

**Figure 5:**
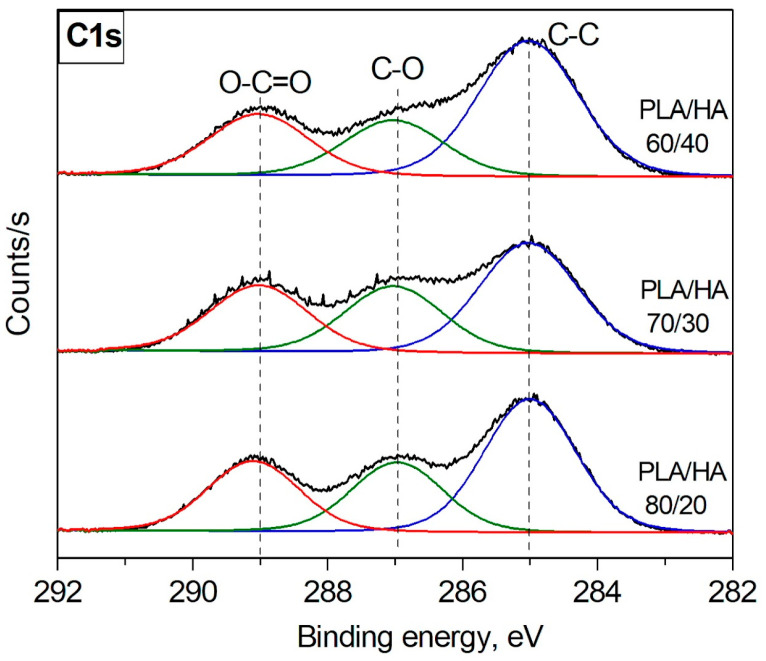
C1s XPS spectra of the untreated PLA/HA composites.

Figure 5 represents the C1s XPS spectra of the PLA/HA composites in their initial state (untreated). In these spectra, the position and shape of the C1s lines correspond to reference data on binding energies for PLA [33]. The content of carbon atoms in the –CH_3_-C, O-CH, and O-C=O combinations is shown in Table 3. The maximum content of the carbon atoms in the –CH_3_-C combination (53.55 at. %) was observed for the initial PLA/HA 80/20 sample. When the fraction of the inorganic component (hydroxyapatite) is increased in the composites, the content of this bond decreases to 48.93 at. % and 45.41 at. % for the PLA/HA 70/30 and PLA/HA 60/40, respectively.

**Figure 6:**
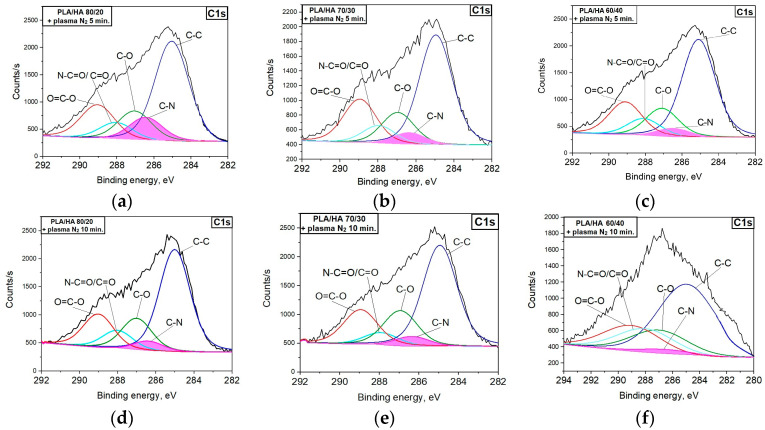
C1s XPS spectra for the composites: (**a**) PLA/HA 80/20; (**b**) PLA/HA 70/30; (**c**) PLA/HA 60/40 after plasma treatment for 5 min; (**d**) PLA/HA 80/20; (**e**) PLA/HA 70/30; (**f**) PLA/HA 60/40 after plasma treatment for 10 min.

Figure 6 shows the C1s XPS spectra for the PLA/HA 80/20, 70/30, and 60/40 composites before and after the nitrogen arc discharge plasma treatments for 5 and 10 min. According to the XPS data (Table 3), the nitrogen plasma treatment leads to a decrease in the content of carbon atoms in the combinations of –CH_3_/C-C (E_b_ = 285.00 eV), O-CH (E_b_ = 286.98 eV) and O-C=O (E_b_ = 289.06 eV) for all the composites up to 40% compared to the initial state. Moreover, after plasma treatment, a new peak with a binding energy of ~286.40 eV was formed, corresponding to the carbon atom in the –C-N combination. The maximum values of the nitrogen-containing bonds were achieved when the composites were treated with plasma for 5 min (Table 3). With the increase in processing time to 10 min, the content of the –C-N bond decreased. In addition, after plasma treatment, N-C=O (E_b_ = 287.90 eV) or C=O-(E_b_ = 288.00 eV) bonds were formed in the surface layer of the composites [34]. The bond energies of these two carbon bonds are quite similar and there is no way to distinguish them, so we will denote them with a slash. The new bond formation processes are always accompanied by the rupture of the existing ones and the redistribution of the atoms that make up the polymer matrix of the composites.

According to the obtained results, the destruction processes of polymer macromolecules predominate in the materials in the plasma treatment conditions. The decrease in the content of carbon atoms in the O-C=O combination is assumed to be associated with the processes of decarbonylation and decarboxylation in the polymer macromolecules [35]. Similar effects due to plasma exposure were observed on fibrous materials based on PLA.

**Figure 7:**
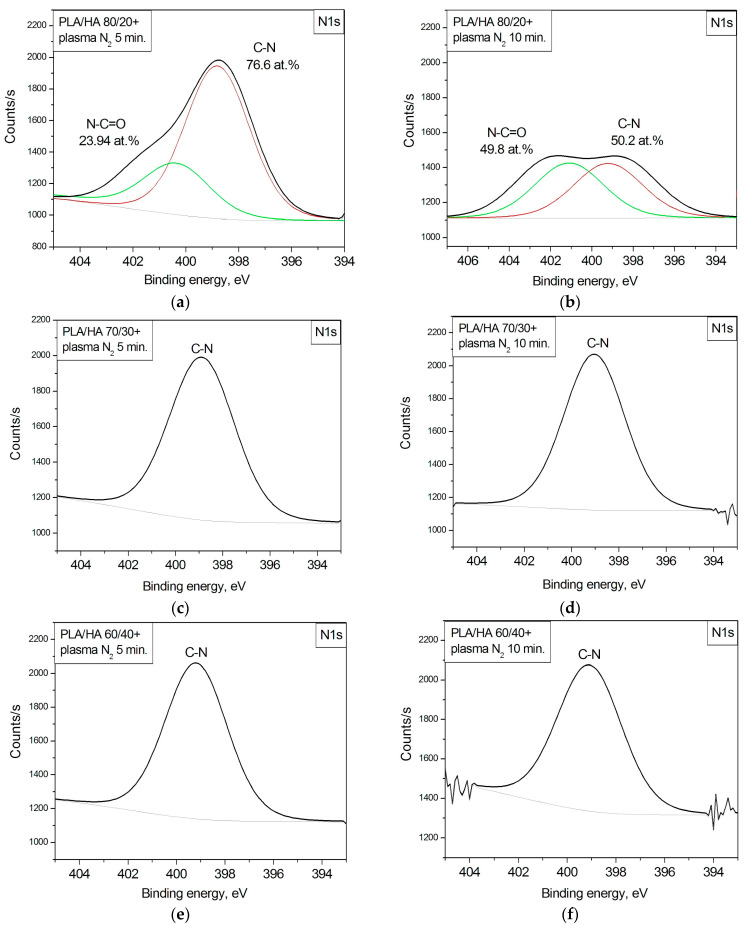
N1s XPS spectra for the composites: (**a**) PLA/HA 80/20; (**b**) PLA/HA 70/30; (**c**) PLA/HA 60/40 after plasma treatment for 5 min; (**d**) PLA/HA 80/20; (**e**) PLA/HA 70/30; (**f**) PLA/HA 60/40 after plasma treatment for 10 min.

Figure 7 shows the N1s XPS spectra for the PLA/HA 80/20, 70/30, and 60/40 composites before and after the nitrogen arc discharge plasma treatments for 5 and 10 min. The formation of the –C-N bond on the surface of all the composites is confirmed by the N1s spectra. The appearance of a nitrogen atom in this combination corresponds to a binding energy of 399.90 eV. In addition, the amide functional group N-C=O was observed in the 5 and 10 min plasma-modified PLA/HA 80/20 samples [33]. This phenomenon can be associated with a larger amount of nitrogen and oxygen atoms on the PLA/HA 80/20 surface than on the surface of the other composites (Table 2) because of the higher content of the polymer component (80 wt.% PLA vs. 70 and 60 wt.% in the other composites), which simplifies the process of chemical bonding of the nitrogen atoms with the substrate atoms.

### 3.2. Surface Morphology of Composite Materials

**Figure 8:**
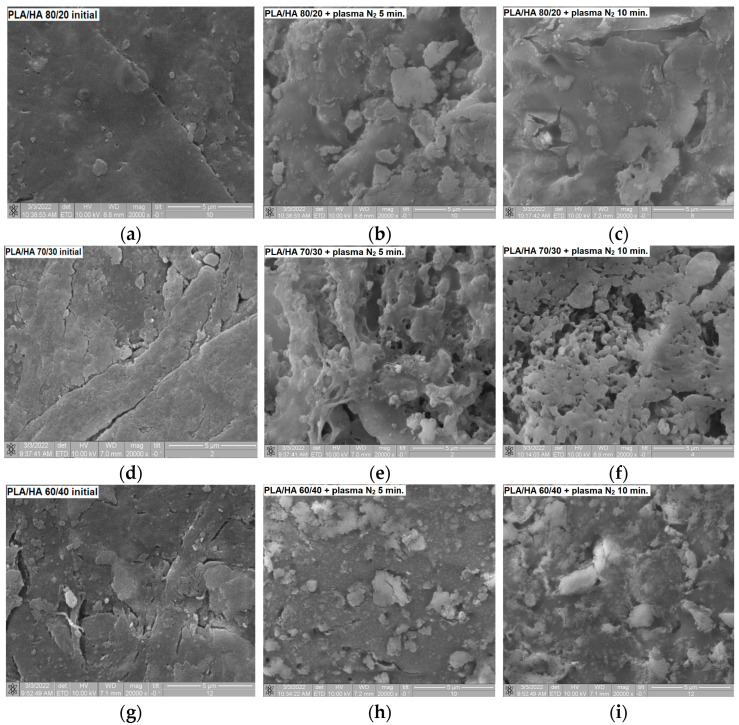
SEM images of the (**a**) initial, (**b)** 5 min plasma-treated, (**c**) 10 min plasma-treated PLA/HA 80/20, (**d**) initial, (**e**) 5 min plasma-treated, (**f**) 10 min plasma-treated PLA/HA 70/30, (**g**) initial, (**h**) 5 min plasma-treated, and (**i**) 10 min plasma-treated PLA/HA 60/40.

The SEM micrographs (Figure 8) reveal that the surface structure of the plasma-treated PLA/HA samples becomes more porous in comparison with the homogeneous structure of the initial samples; protrusions and pores appear. These changes in morphology can be caused by the knocking out of sections of the composite by the chaotic movement of charged particles in the plasma volume, accompanied by the formation of inhomogeneities on the surface.

### 3.3. Wettability of PLA/HA Composite Materials

**Figure 9:**
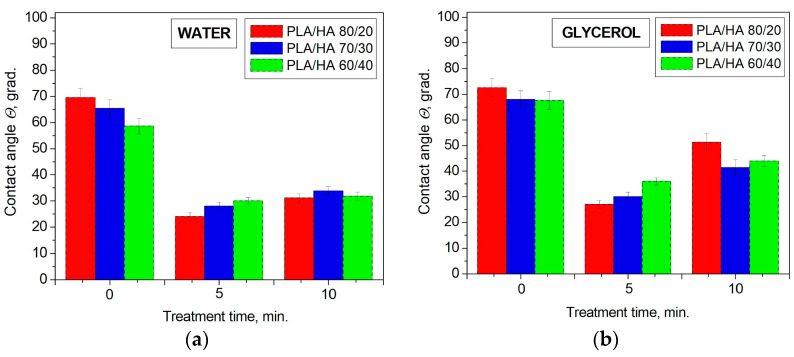
Contact angles of PLA/HA composites before and after nitrogen plasma treatment wetted with (**a**) water and (**b**) glycerol.

Changes in the chemical and elemental composition of the surface of the PLA/HA composites caused by the nitrogen plasma treatment affect their wettability characteristics. Wettability is known to play an important role in biochemical processes occurring at the bone tissue–liquid interface of a living organism [36]. The contact angles for the untreated composites are equal: 70° and 73° for the PLA/HA 80/20; 65° and 68° for the PLA/HA 70/30; and 59° and 68° for the PLA/HA 60/40 when wetted with water and glycerol respectively. According to the data (Figure 9), the contact angles of the composites tended to decrease after the nitrogen plasma treatment for 5 min. However, when the plasma processing time increased to 10 min, the contact angle values increased slightly relative to the 5 min treatment but did not reach the contact angles of the initial materials.

The minimum contact angles in contact with water (24°) and glycerol (30°) correspond to PLA/HA 80/20 after the plasma treatment under nitrogen flow for 5 min. It should be noted that after plasma modification, the contact angle for all the composites in contact with two liquids remained less than 90°, which means that the materials retained their hydrophilic properties. Moreover, the decrease in contact angles after plasma treatment indicates an increase in the hydro- and oleophilicity of the surface of the PLA/HA composites.

**Figure 10:**
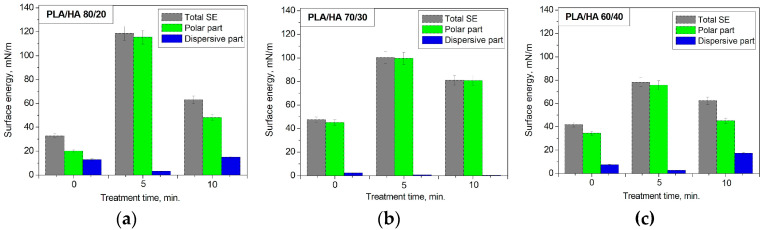
Surface energy dependence on the nitrogen plasma treatment duration for PLA/HA (**a**) 80/20, (**b**) 70/30, and (**c**) 60/40.

According to the results obtained (Figure 10), the nitrogen arc discharge plasma treatments for 5 and 10 min lead to the increase of the surface energy of the composite materials relative to the initial samples. In this case, an increase in the total surface energy is accompanied by an increase in its polar component and a decrease in the dispersion component. It was shown that after the nitrogen plasma treatment for 5 min, the total surface energy of the PLA/HA 80/20 notably increased from 32.73 to 118.55 mN/m; of the PLA/HA 70/30, from 47.38 to 100.19 mN/m; and of the PLA/HA 60/40, from 41.76 to 78.17 mN/m. The plasma treatment time increased up to 10 min resulted in the total surface energy decrease with a simultaneous decrease in the polar and dispersive components (Figure 10).

**Figure 11:**
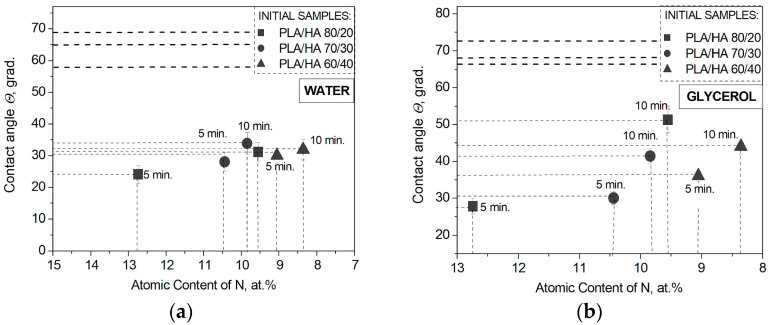
(**a**) Water and (**b**) glycerol contact angle dependence on the nitrogen atomic concentration in the PLA/HA composites’ surface layer.

We assume that a decrease in the PLA/HA contact angles with water and glycerol after plasma modification may be associated with the accumulation of nitrogen atoms [N, at. %] in the free and bound states (–C-N bond) in the surface layer (Table 2 and Table 3). As can be seen in Figure 11, the maximum atomic concentrations of nitrogen, corresponding to the minimum contact angle values, are observed when the composites were plasma-treated for 5 min. With a further increase in the treatment duration to 10 min, the nitrogen concentration decreases, and the contact angle values slightly increase for all the composites. This is probably due to a certain limit of surface saturation with nitrogen, which, upon reaching, the already introduced atoms are knocked out.

**Figure 12:**
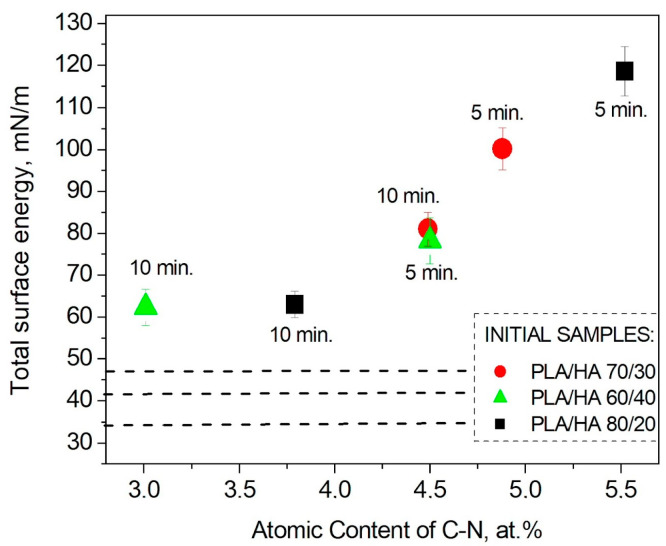
Dependence of the PLA/HA samples’ total surface energy on the content of the –C-N bond in the composite surface layer after nitrogen plasma treatment.

The dependence of free surface energy on the surface chemical composition of the plasma-modified composites was revealed. The formation of the –C-N bond was established to enhance the surface energy of the PLA/HA composites. The maximum content of the –C-N bond corresponds to the highest value of surface energy for all the types of composite after plasma treatment for 5 min (Figure 12). When the treatment duration increased to 10 min, the content of the –C-N bond decreased relative to the 5 min plasma exposure, and the free surface energy values for all the composites also decreased. Presumably, when treated with plasma for 5 min, the PLA/HA surface is saturated with nitrogen atoms (the concentration of nitrogen atoms reaches a threshold value), and a further increase in the treatment duration leads to the destruction of both the original polymer bonds in the composites and the rupture of the newly formed –C-N bonds. The decrease in the content of the –C-N bond, which is a polar functional group, leads to a decrease in surface energy, although its values remain higher than those for the initial non-plasma-treated composites (Figure 12). The decrease in the contact angles and the enhancement of the surface energy means that the surface of the modified PLA/HA samples becomes more wettable upon contact with liquids, probably due to the oxidative processes occurring in the surface layers under the nitrogen plasma treatment conditions [37].

### 3.4. Effect of PLA/HA Composites on Macrophage Viability 

Composite materials used to fill bone defects can lead to negative effects, such as inflammation. Macrophages are one of the main participants in inflammatory processes that can regulate them. For this reason, monocyte-derived macrophages were chosen as a model system to study the biocompatibility of the composites [38].

**Figure 13:**
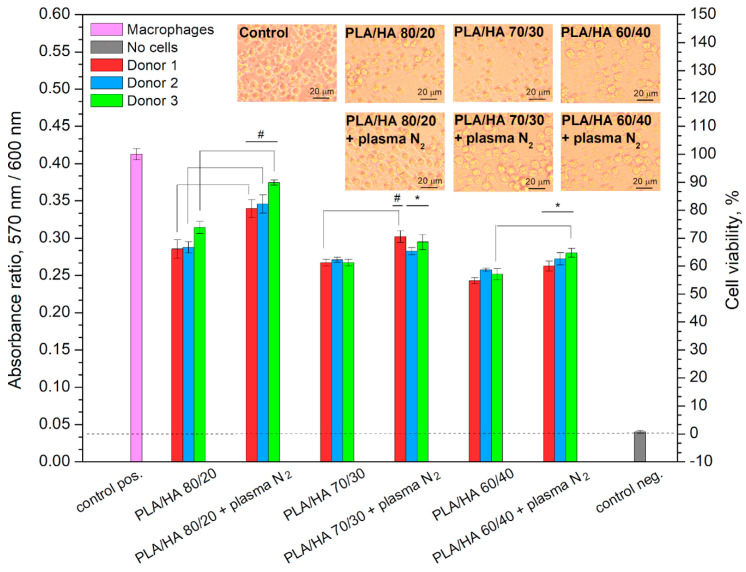
Macrophage viability in the presence of PLA/HA composites. Differences between unmodified and modified composites for each donor were noted; (*) existing differences from PLA/HA 80/20 composites are noted; (#) existing differences from PLA/HA 60/40 composites are noted (at *p* < 0.05). Magnification in photographs ×100.

Macrophage viability was assessed in the presence of the initial composites and the 5 min nitrogen plasma-treated PLA/HA. The results of the study are presented in Figure 13. For all the samples, a decrease in the viability of macrophages was observed compared to the control (the cells cultured without the materials). In the presence of the untreated composites, lower rates of macrophage viability were observed than in the presence of the treated ones: 66–74% for PLA/HA 80/20; 61–62% for PLA/HA 70/30; and 55–58% for PLA/HA 60/40. Significant differences between the number of living cells on the unmodified and modified surfaces were mainly observed for the PLA/HA 80/20. For the PLA/HA 60/40 and PLA/HA 70/30, the differences in viability were significant only for one donor out of three.

The 5 min nitrogen plasma-modified PLA/HA 80/20 demonstrates the best results, as the cell survivability is in the range of 81–90%. In the cases of the modified PLA/HA 70/30 and PLA/HA 60/40, it does not exceed 70% and 65%, respectively. It can be supposed that the more the inorganic component (hydroxyapatite) in the composite content, the fewer number of cells can survive in the presence of the composites.

The obtained results of the cells’ viability are consistent with the data of the elemental composition, wettability, and surface energy of the PLA/HA composites before and after the plasma treatment. The PLA/HA 80/20 treated with nitrogen plasma for 5 min is characterized by the highest content of –C-N bond and the lowest contact angle with polar liquids. It was shown [28] that the plasma-treated PLA scaffolds with functional nitrogen-containing groups in the surface layer have a positive effect on cell survival compared to the initial PLA. A biocompatibility improvement of polylactic acid after radiofrequency nitrogen plasma surface treatment was also noted in [39].

It is known that chemical composition and wettability are among the main surface characteristics of the implantology-used materials that influence interactions with cells. A protein layer is required as an intermediary for the cell to contact the surface of the implant. In turn, proteins can be adsorbed from biological fluid, interacting with special functional groups on the surface (hydroxyl, methyl, amino groups, etc.) [40]. In this study, the formation of polar nitrogen-containing groups on the surface of the modified composites is suggested, which may be the reason for increasing the water wettability of the composites and allowing the interaction with proteins and cells. We have demonstrated that the number of living cells in the presence of the modified composites decreases along with a decrease in the content of –C-N bonds and a weakening of the hydrophilic properties.

## 4. Conclusions

The physicochemical and functional properties of PLA/HA composite materials in mass ratios of the components of 80/20, 70/30, and 60/40 with a nitrogen arc discharge plasma-modified surface were studied. According to the obtained results, the plasma exposure was found to change the surface chemical composition of the composites. The processes of the destruction of bonds and redistribution of atoms that make up the polymer matrix of the composites took place, accompanied by the formation of new polar functional oxygen- and nitrogen-containing groups. The formation of the –C-N and N-C=O/-C=O bonds improves the wettability of the PLA/HA surface; for instance, the free surface energy of the PLA/HA 80/20 increased from 33 to 119 mN/m. The presence of polar functional groups on the surface of the PLA/HA composites can also ensure interaction with cells (macrophages). It has been shown that the formation of a –C-N bond and an increase in surface hydrophilicity positively affect the biocompatibility of the plasma-treated composites compared to the unmodified materials.

We assume that the alteration of the physicochemical characteristics of the composites under plasma exposure occurs primarily due to changes in the polymer component (polylactic acid), while the inorganic part (hydroxyapatite) remains inert to the plasma treatment. The obtained results indicate similar effects of plasma exposure under similar conditions on both the PLA/HA composites and the PLA scaffolds, which we have previously studied. 

Thus, the dependencies of alteration of the physicochemical properties and biocompatibility of the PLA/HA composite materials under the plasma treatment conditions have been established, and the optimal parameters of controlled plasma processing have been selected to predict the functional properties of polylactic acid-based materials. The fundamental principles for obtaining promising materials with the required properties for eliminating bone defects have been created.

## Figures and Tables

**Table 1:** Plasma treatment conditions.

**Working gas**	N_2_	
**Discharge**	Arc	
**Pressure in the working chamber, Pa**	0.3	
**Plasma exposure time, min**	5	10
**Discharge current, A**	5	
**Temperature in the chamber, °C**	30	46
**Energy of particles in plasma, eV**	5	8

**Table 2:** Atomic [C, at. %]/[O, at. %] and [Ca, at. %]/[P, at. %] ratios of PLA/HA 80/20, 70/30, 60/40, and pure HA before and after plasma treatment; atomic content of nitrogen after plasma treatment.

Sample	[C, at. %]/[O, at. %]	[Ca, at. %]/[P, at. %]	Atomic Content of N, at. %
PLA/HA 80/20 initial	1.10 ± 0.2	1.85 ± 0.2	-
PLA/HA 80/20 + plasma N_2_ (5 min)	0.98 ± 0.3	1.79 ± 0.1	12.74 ± 0.3
PLA/HA 80/20 + plasma N_2_ (10 min)	0.85 ± 0.3	1.72 ± 0.2	9.55 ± 0.1
PLA/HA 70/30 initial	1.10 ± 0.1	1.75 ± 0.2	-
PLA/HA 70/30 + plasma N_2_ (5 min)	0.94 ± 0.3	1.71 ± 0.2	10.44 ± 0.1
PLA/HA 70/30 + plasma N_2_ (10 min)	0.95 ± 0.3	1.67 ± 0.1	9.84 ± 0.1
PLA/HA 60/40 initial	0.90 ± 0.3	1.63 ± 0.2	-
PLA/HA 60/40 + plasma N_2_ (5 min)	0.72 ± 0.3	1.59 ± 0.1	9.05 ± 0.3
PLA/HA 60/40 + plasma N_2_ (10 min)	0.73 ± 0.3	1.68 ± 0.2	8.36 ± 0.1
HA initial	-	1.77 ± 0.1	-
HA + plasma N_2_ (5 min)	-	1.79 ± 0.1	-
HA + plasma N_2_ (10 min)	-	1.75 ± 0.3	-

**Table 3:** Positions of C1s lines and contents of bonds in PLA/HA 80/20, 70/30, 60/40.

Sample	Binding Energy, eV				
	285.00	286.98	289.06	288.00	286.40
	Content of Bonds in C1s Spectrum, at. %				
	–CH_3_/C-C	–CH-O	O-C=O	N-C=O/C=O	–C-N
PLA/HA 80/20 initial	53.5 ± 0.2	22.1 ± 0.6	24.3 ± 0.2	-	-
PLA/HA 80/20 + plasma N_2_ (5 min)	42.6 ± 0.2	21.4 ± 0.7	23.8 ± 0.3	6.7 ± 0.7	5.5 ± 0.6
PLA/HA 80/20 + plasma N_2_ (10 min)	41.1 ± 0.3	16.0 ± 0.6	22.0 ± 0.6	17.1 ± 0.7	3.8 ± 0.4
PLA/HA 70/30 initial	48.9 ± 0.6	25.4 ± 0.7	25.6 ± 0.3	-	-
PLA/HA 70/30 + plasma N_2_ (5 min)	42.3 ± 0.3	22.1 ± 0.6	24.1 ± 0.6	6.4 ± 0.7	4.8 ± 0.9
PLA/HA 70/30 + plasma N_2_ (10 min)	42.5 ± 0.2	18.5 ± 0.3	25.0 ± 0.3	9.5 ± 0.6	4.5 ± 0.2
PLA/HA 60/40 initial	45.4 ± 0.4	27.3 ± 0.3	27.2 ± 0.2	-	-
PLA/HA 60/40 + plasma N_2_ (5 min)	44.9 ± 0.6	15.9 ± 0.2	26.3 ± 0.7	8.4 ± 0.2	4.5 ± 0.7
PLA/HA 60/40 + plasma N_2_ (10 min)	41.9 ± 0.4	16.0 ± 0.2	22.0 ± 0.2	17.1 ± 0.7	3.0 ± 0.4

## Data Availability

The data that support the findings of this study are available. All the data generated or analyzed during this study are included in this published article.
